# Economic Empowerment Interventions on Mental Health Outcomes Among People Living with HIV in Low and Middle-Income Countries: A Systematic Literature Review

**DOI:** 10.3390/bs16091680

**Published:** 2026-09-17

**Authors:** Peter Onchuru Mokaya, Evans Kasmai Kiptulon, Dahabo Adi Galgalo, Mohammed Elmadani, Gabriella Hideg-Fehér

**Affiliations:** 1 Doctoral School of Health Sciences, Faculty of Health Sciences, University of Pécs, 7622 Pécs, Hungary; evans.kasmai.kiptulon@pte.hu (E.K.K.); galgalo.dahabo@pte.hu (D.A.G.); elmadani88@gmail.com (M.E.); 2 Faculty of Health Sciences, Institute of Physiotherapy and Sport Science, University of Pécs, 7622 Pécs, Hungary; gabriella.hideg@etk.pte.hu

**Keywords:** HIV, economic empowerment Intervention, low and middle-income countries, mental health

## Abstract

There is strong evidence of the correlation between economic hardship and poor mental health outcomes among people living with HIV (PLHIV). However, the causal efficacy and mechanisms of specific economic interventions through which they contribute to mental health improvement among PLHIV remain fragmented and inconsistent. This systematic review aims to collate and synthesize existing evidence on the effect of economic empowerment interventions on mental health symptoms among PLHIV in low- and middle-income countries (LMICs). We conducted a comprehensive literature review that adhered to the PRISMA guidelines for methodological rigor and transparency. Comprehensive searches were conducted in PubMed, Web of Science, Embase, CINAHL, Scopus, and MEDLINE. We conducted a narrative synthesis of 11 studies: two cohorts, two qualitative, one intervention (non-randomized), and six randomized clinical trials. We registered this review with PROSPERO (CRD420251152471). The process theory served as an analytical framework. Evidence suggests that multi-component economic empowerment interventions (e.g., combined savings, skills training, and psychological support) may be associated with improved mental health among PLHIV in LMICs. Qualitative evidence from other studies suggested that reduced financial stress and strengthened psychological resources may contribute to improved mental health. The effectiveness of the intervention depends not only on simple cash transfers but also on a theoretically sound integrated approach. Interventions were more effective among lower-income groups. Integration of multi-component economic and psychosocial interventions into standard HIV care could improve mental health outcomes among PLHIV in LMICs and is hereby recommended by this study.

## 1. Introduction

The intersection of economic burden and mental health among people living with HIV in low- and middle-income countries is an important public health challenge that requires further investigation.

People living with HIV disproportionately face mental health issues due to the synergistic effects of stigma, health-related challenges, and poverty. Economic difficulties may contribute to poorer health outcomes among people living with HIV. These difficulties may lead to limiting healthcare access, fostering hopelessness, reducing treatment adherence, and consequently leading to high viral loads and low retention rates (Lund et al., 2010; Nakimuli-Mpungu et al., 2021; Van Doren et al., 2026).

This review is based on the process theory, which posits that interventions create pathways that mediate change (Moore et al., 2015). In this study, we hypothesize that economic empowerment intervention forms a pathway through which mental health improves by enhancing self-concept and hope through the development of economic and psychological resources among people living with HIV in low and middle-income countries. Structural economic interventions, such as cash transfers, microfinance support, livelihood initiatives, agricultural assistance, and savings loans, play a critical role in bridging the poverty gap, which is a root cause of physical and mental health problems (Gibbs et al., 2017; Kennedy et al., 2014).

There is strong evidence of the correlation between economic hardship and poor mental health outcomes among people living with HIV. However, the causal efficacy and mechanism of specific economic interventions through which they contribute to mental health improvement remain fragmented with inconsistent findings (Kennedy et al., 2014; Stoner et al., 2021). For example, while some studies indicate that cash transfers can reduce mental health symptoms (Guimarães et al., 2023; Van Doren et al., 2026), other studies yield mixed results (Kennedy et al., 2014; Nadkarni et al., 2019).

Again, several previous reviews have examined related topics in low- and middle-income countries. A scoping review of Conteh et al. (2023) mapped the effectiveness of integrating mental health in HIV programs. They concluded that integrating mental health services into HIV care contributes to the improvement of the diagnosis and treatment of mental disorders related to substance abuse in PLHIV. Similarly, a scoping review by Nwogwugwu et al. (2025) that aimed at collating existing evidence on mental health interventions for people living with HIV/AIDS in Africa concluded that incorporating mental health services among people living with HIV improves health outcomes. A systematic review by the Lancet Child & Adolescent Health series examined mental health care integration for adolescents living with HIV, noting the potential of economic interventions but calling for more evidence (Cluver et al., 2022).

Our review advances this literature in three key ways. First, it focuses explicitly on mental health outcomes as primary endpoints, whereas previous reviews have focused on HIV care outcomes (e.g., adherence, viral suppression) with mental health as secondary. Second, it systematically distinguishes between multi-component economic–psychosocial interventions and cash transfer-only interventions, providing comparative insights for program design. Third, it integrates quantitative and qualitative evidence within a process theory framework to elucidate mechanisms of change, addressing gaps identified in previous reviews. To our knowledge, this is the first systematic review to specifically synthesize evidence on the effectiveness of economic empowerment interventions for mental health outcomes among PLHIV in LMICs, and to distinguish intervention types by their theoretical mechanisms.

**Figure 1:**
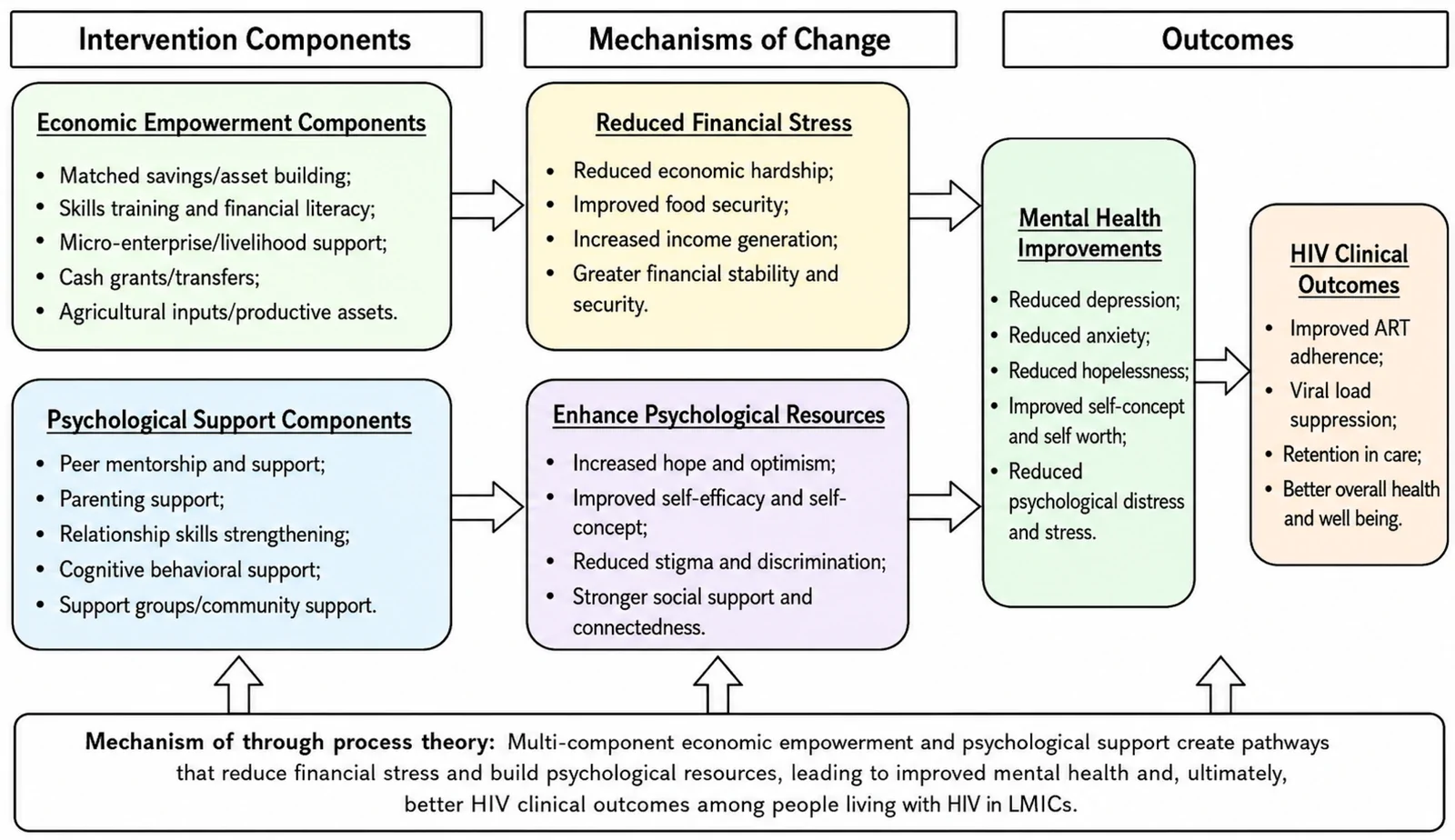
Conceptual framework illustrating pathways from economic empowerment interventions to mental health outcomes among PLHIV in LMICs.

Figure 1 presents the proposed conceptual framework of our study. Grounded in process theory Moore et al. (2015), the framework posits that economic empowerment interventions activate two interconnected mediating pathways. It is hypothesized that these pathways lead to reduced financial stress through improved economic stability and food security, and enhanced psychological resources through increased hope, self-concept, and reduced stigma. These pathways converge to improve mental health outcomes, which in turn facilitate HIV clinical outcomes.

This systematic review aimed to collate and synthesize existing evidence on the effect of economic empowerment interventions on mental health outcomes among people living with HIV in low- and middle-income countries. This review was guided by a primary research question: “What is the effectiveness of economic empowerment intervention on mental health outcome among people living with HIV in low- and middle-income countries?” This review aimed to explore different types of economic empowerment interventions that have been studied, most mental health outcomes influenced by these interventions, and reported mechanisms of change and review moderating factors such as age, gender, intervention duration among others.

In our study, structural economic empowerment intervention involves projects that aim to modify socioeconomic structures through capacity building, creation of economic opportunities, and strengthening the social protection systems for PLHIV in LMICs. This can be achieved through the capacity building of individuals, supporting them in establishing economic support institutions, such as self-help groups, reforming existing institutions, and creating inclusive economic opportunities, as well as forming or strengthening social protection systems. The goal of such interventions is to address the fundamental economic and social factors that influence HIV vulnerability and treatment outcomes (Cluver et al., 2015; Evans et al., 2010; Gurnani et al., 2011; Iskarpatyoti et al., 2018; Van Der Wal et al., 2021).

## 2. Methods

### 2.1. Study Design

Our design was guided by the aim of understanding the relationship or effect of economic intervention on mental health outcomes among PLHIV in LMICs. We conducted a comprehensive literature review that followed the PRISMA guidelines for methodological rigor and transparency (Nakimuli-Mpungu et al., 2021; Shamseer et al., 2015). We included both quantitative and qualitative studies employing quasi-experimental, randomized clinical trials, and cohort studies that were published between January 2016 and January 2026. While observational studies demonstrate the association between economic hardship and poor mental health, this review focused exclusively on intervention studies to establish causal efficacy and find effective program components.

To minimize publication bias and enhance comprehensiveness, we searched both peer-reviewed and grey literature in our study. Due to the heterogeneity of the population, interventions, assessment tools, and mental health outcomes, we conducted a narrative synthesis analysis (Shamseer et al., 2015; Taylor et al., 2021).

### 2.2. Condition or Domain Being Studied

This review focused on the intersection of economic hardship, HIV, and mental health in LMICs. People living with HIV are disproportionately affected by mental disorders, which are exacerbated by poverty and economic hardships. The effect of HIV on mental health has become a public health concern in LMICs (Bernard et al., 2017; Gacau et al., 2024; Mwangala et al., 2022; Nyongesa et al., 2021). Economic hardship and the incidence of HIV have a bidirectional association in LMICs (Katana et al., 2020; Tsai et al., 2012). This review focuses on how economic empowerment interventions, such as cash transfers, livelihood initiatives, agricultural programs, and microfinance interventions that can disrupt poverty-related mental health challenges such as depression, anxiety, and psychological distress among people living with HIV in LMICs.

### 2.3. Eligibility Criteria

**Population:** Adolescents and adults living with HIV in low- and middle-income countries. In a mixed population (PLHIV and HIV-negative individuals) only data from the subgroup of people living with HIV were extracted and analyzed.

**Intervention:** Economic intervention/empowerment, e.g., cash transfers, microfinance interventions, livelihood programs, agricultural interventions, savings and loan group activities among others.

**Comparison:** People living with HIV receiving no economic intervention, receiving usual care or other alternative non-economic support interventions.

**Primary outcomes:** Mental health symptoms (e.g., depression, anxiety, psychological distress, general mental health symptoms) measured via validated tools. Secondary outcomes: adherence to antiretroviral therapy, viral load suppression, and retention.

**Study types to be included:** Randomized and non-randomized clinical trials, and cohort studies.

### 2.4. Search Strategy

Comprehensive searches were conducted in PubMed, Web of Science, Embase, CINAHL, Scopus, and MEDLINE. Grey literature was thoroughly searched, and relevant referenced studies screened. Included studies were restricted to peer-reviewed publications in English from the past 10 years in low- and middle-income countries. Our study focused on keywords and synonyms of mental health,

HIV and AIDS, and economic empowerment as elaborated below. Boolean operators AND/OR were utilized to ensure precision and inclusivity.

**Mental Health:** “Mental health” OR “Psychological well-being” OR “Mental well-being” OR “Cognitive health” OR “Behavioral health” OR “Psychological health” OR “Emotional health” OR “Psychosocial health” OR “Inner well-being” OR “State of mind” OR “Psychiatric health” OR “Mental status” OR “Neuropsychiatric condition” OR “Psychological functioning” OR “Psychopathology” OR “Mental state” OR “Psychosocial outcomes” OR “Mental health outcomes” OR “Psychological resilience” OR “Emotional regulation” OR “Quality of life” OR “Subjective well-being” OR “Social-emotional health” OR “Mental illness” OR “Mental disorder” OR “Emotional distress” OR “Psychological distress” OR “Mood disorder” OR “Anxiety disorder” OR “Psychiatric condition” OR “Depression” OR “Anxiety”

**AND**

**HIV and AIDS:** “HIV” OR “Human Immunodeficiency Virus” OR “HIV/AIDS” OR “AIDS” OR “PLHIV” OR “People living with HIV” OR “HIV-positive” OR “HIV-infected individuals” OR “HIV patients” OR “HIV-affected communities” OR “HIV seropositive” OR “HIV+”

**AND**

**Economic Empowerment:** “Economic empowerment” OR “Livelihood programs” OR “Income-generating activities” OR “Cash transfer” OR “Cash transfer program” OR “Conditional cash transfer” OR “Unconditional cash transfer” OR “Microfinance” OR “Microcredit” OR “Vocational training” OR “Economic strengthening” OR “Savings group” OR “Asset-building” OR “Financial inclusion” OR “Self-help groups” OR “Entrepreneurship program” OR “Income support”

### 2.5. Screening of the Article

Study selection began with screening titles and abstracts, followed by full-text reviews by two independent reviewers for eligibility. Secondly, the full text was reviewed to confirm that the selected articles conformed with our eligibility criteria. Where possible, discrepancies were resolved through consensus or by the senior researcher in our team (Conteh et al., 2023). To ensure reproducibility and minimization of bias, screening was done using the Covidence software package (Covidence, 2026). This approach adhered to the synthesis without meta-analysis reporting guidelines of Campbell et al. (2020), which we followed to ensure transparency in our narrative synthesis methods.

### 2.6. Data Extraction

Based on the objectives and study questions, we came up with a pre-tested data extraction tool. This tool was used by two independent reviewers to extract data from the included studies. Extracted data included: the authors, year of publication, study location, study objectives, study design, intervention details, sample size, population baseline and endline sociodemographic characteristics, tools of assessment, outcome effect size, summary of primary outcomes, summary of secondary outcomes (Conteh et al., 2023).

### 2.7. Quality Appraisal and Risk of Bias of the Included Studies

Two independent reviewers assessed the methodological quality of randomized studies using the Cochrane Risk of Bias 2 (RoB2) tool (Sterne et al., 2019), non-randomized studies using Risk of Bias In Non-randomized Studies-of Intervention (ROBINS-I) tool (Sterne et al., 2016), and Critical Appraisal Skills Programme (CASP) tool for qualitative studies (CASP, 2024). Because these tools assess different methodological constructs, their results were reported separately and were not combined into a single overall certainty rating.

This exercise identified potential sources of bias, including confounding, selection bias, outcome misclassification, and other sources. A senior researcher in the team resolved any discrepancies that arose during this exercise. This exercise helped us to assess the overall confidence in this study.

Lower-risk of bias studies consistently favored multi-component interventions; cash-only evidence was mixed. Effects were concentrated among adolescents, low-asset participants, and women, suggesting effect modification by vulnerability level. The heterogeneity of study design, tools, outcomes prevented us from coming up with a single confidence rating. We conducted a sensitivity analysis to lower risk studies and qualitative studies which confirmed the direction and consistency of our core findings. The summary outcome of quality appraisal can be found in Table 1.

### 2.8. Strategy for Data Synthesis and Analysis

Due to heterogeneity, we conducted a narrative synthesis. We avoided conducting a meta-analysis. Narrative analysis allowed for a systematic, thematic exploration of the evidence, enabling us to compare and contrast findings across different intervention models, identify common mechanisms of action, and elucidate contextual factors influencing effectiveness, which aligns with the exploratory aims of this review. Synthesis followed a four sequential iterative stages that followed preliminary synthesis, exploration of the relationship between and within studies, assessment of synthesis robustness, and interpretation of findings. Following the synthesis without meta-analysis (SWiM) reporting guidelines, we did a weaving approach in combining the evidence of quantitative with qualitative research designs. This integrated narrative synthesis aided in assessing whether interventions worked (quantitative) while qualitative findings explained how and why (Campbell et al., 2020).

The study objective and research questions also guided the synthesis of the mined data. We grouped the evidence based on the intervention types and mental health outcomes. Subgroup analysis was conducted to explore the effect of the intervention in different groups and settings. Due to the heterogeneity in intervention (e.g., cash transfers versus integrated community livelihood programs), mental health assessment tools (e.g., Child Depression Inventory, Beck’s Hopelessness Scale, Hopkins Symptom Checklist-25 (HSCL-25), Patient Health Questionnaire-2, Generalized Anxiety Questionnaire 2, Center for Epidemiologic Studies Depression Scale), different mental health outcomes (e.g., depression, anxiety, and hopelessness), different target population demographics (e.g., adolescents, adults, men, women), and study design (RCT, cohort and non-randomized) meta-analysis was not feasible. Two researchers independently coded the data, then debated and agreed on the final themes to ensure the quality and reliability of the content analysis. They organized studies by characteristics, intervention type (Multi-Component Economic Programs such as Savings, Training, Support; Pure Economic Interventions such as Cash Transfers Only), mental health outcome domain, effect estimates, and reported mediators. We used tabulation and grouping to organize studies by intervention category and outcome domain. Descriptive summaries were developed for each study and verified by a third reviewer (Appendix A Table A1).

### 2.9. Registration

**Figure 2:**
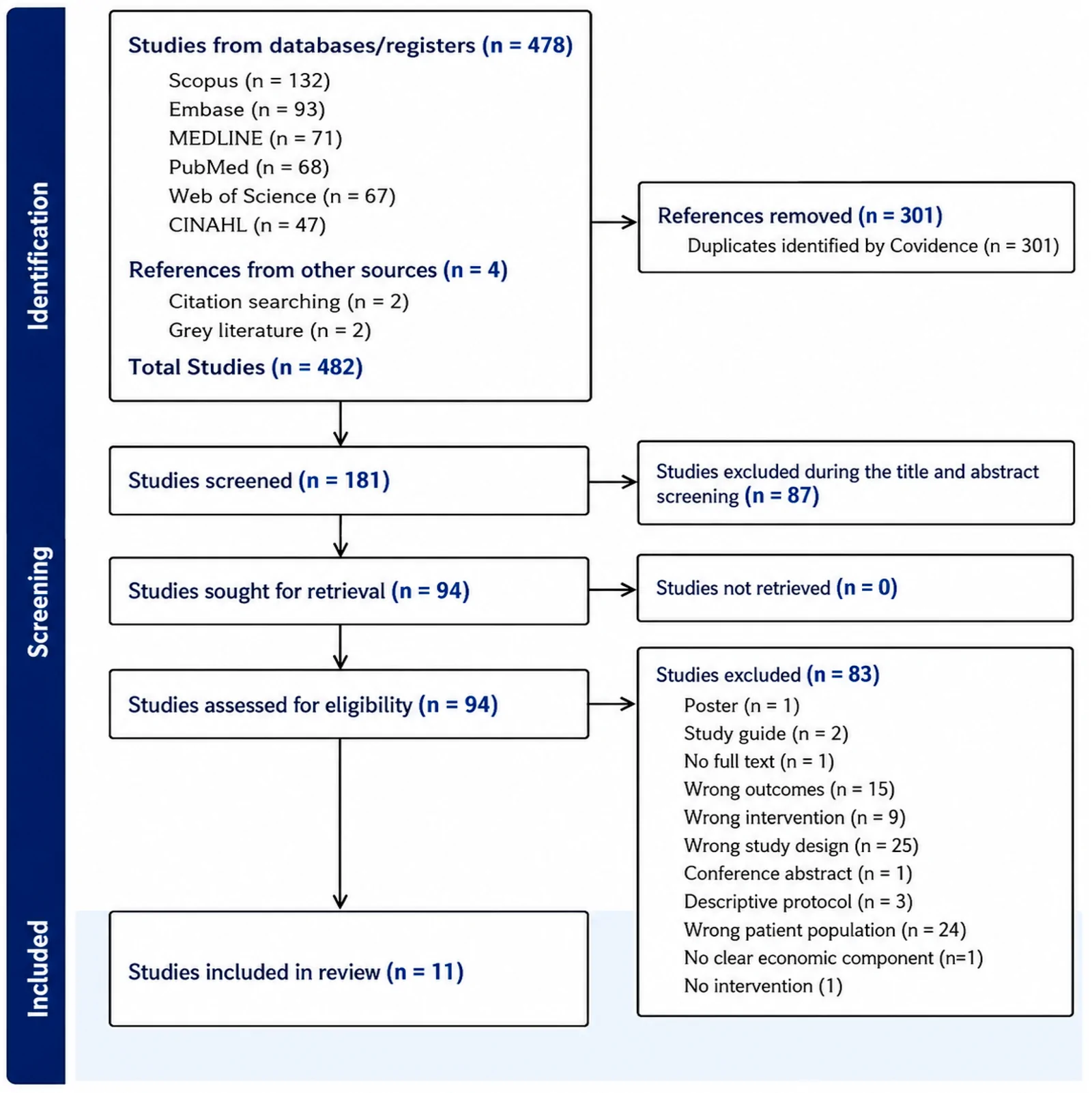
PRISMA flow diagram of included and excluded articles.

On 21 September 2025, we registered this review with the registration number CRD420251152471 in the International Prospective Register for Systematic Reviews (PROSPERO) worldwide register. The Preferred Reporting Item for Systematic Reviews and Meta-Analyses (PRISMA) statement’s guidelines were followed in this review. The PRSIMA flow of the study selection process can be found in Figure 2.

## 3. Results

Synthesis of the evidence from 11 studies: two cohorts, two qualitative, one intervention (non-randomized), and six randomized clinical trials across low- and middle-income countries was done (Table 2 and Table 3). We searched grey literature, but only peer-reviewed studies met final eligibility criteria.

### 3.1. Sensitivity Analysis and Exploration of Heterogeneity

In this study, the sensitivity analyses demonstrated robustness of the core finding that multi-component economic–psychosocial interventions improve mental health outcomes among PLHIV in LMICs. The pattern remained consistent when restricting analyses to six lower-risk studies (Cavazos-Rehg et al., 2021; Chi et al., 2025; Cluver et al., 2019; Kizito et al., 2025; Shimizu et al., 2016; Van Doren et al., 2026). Because different appraisal tools assess different methodological constructs, we did not derive a single cross-design certainty rating. Findings should therefore be interpreted according to the appraisal appropriate for each study design.

Findings were directionally consistent across RCTs and non-randomized designs. Effect detection varied by measurement approach, with continuous scales (CES-D, PHQ-9, HSCL-25) being more sensitive to change than dichotomous thresholds. Patterns of heterogeneity were identified through narrative comparison of intervention characteristics and study findings rather than formal quantitative subgroup analyses. Multi-component interventions consistently indicated positive effects; cash transfer-only interventions showed mixed results. This pattern was consistent across study designs and settings. However, as we did not conduct a meta-analysis, this observation reflects a narrative judgment based on the pattern of findings across studies rather than a formal quantitative subgroup test.

Effects were concentrated among adolescents, low-asset participants, and women, suggesting effect modification by vulnerability level (Campbell et al., 2020).

Evidence from different studies across multiple countries demonstrated the causal efficacy of economic empowerment interventions on mental health outcomes as seen in Table 2 and Table 3 and Appendix A Table A1 and Table A2. Results are presented in segments of multi-component programs, pure cash transfer interventions, and the mechanisms of action.

### 3.2. Multi-Component Economic Programs (Savings + Training + Support)

Multi-component interventions that combined economic interventions and psychosocial or structural support programs were associated with mental health benefits compared to standalone interventions. Cohort studies from South Africa and Zambia (Table 3) indicate that integrated models involving structured economic interventions lead to better mental health outcomes. This review found that the RCT of the KHANA livelihood program in Cambodia (Table 2), combining village savings/loans, skills training, and cash grants, reduced odds of depressive symptoms by 32% (Shimizu et al., 2016).

A pilot RCT in Malawi of the “Mlambe” intervention included 10 monthly sessions on economic empowerment and relationship skills, plus incentivized joint savings accounts. This study showed significant reductions in depression (Cohen’s d = 0.41–0.46), stress (d = 0.52–0.54), and hopelessness (d = 0.26–0.29) compared to enhanced usual care, which included brief alcohol counseling and standard HIV care. Women experienced significantly greater reductions in stress and hopelessness than men (Van Doren et al., 2026).

In Uganda, a cluster RCT intervention of the Suubi + Adherence project (Table 2) that matched child development accounts, financial literacy training, and peer mentorship among adolescents living with HIV significantly improved latent mental health outcomes (shared variance of depressive symptoms, hopelessness, and self-concept) at month 36 (B = −0.45, *p* < 0.05). Family assets and employment significantly mediated this effect by explaining 42–72% of the total effect across follow-ups. This indicates that significant effects were amplified among the most vulnerable adolescents (Cavazos-Rehg et al., 2021).

Under the same RCT study (Suubi + Adherence) that tested the effect of economic empowerment on viral suppression and mental health (depressive symptoms, hopelessness, and self-concept) showed no effect on the full sample for any mental health outcomes (Table 2). A secondary, pre-specified analysis of the same Ugandan RCT revealed important subgroup effects. While the intervention showed no significant effect on mental health outcomes in the full sample, it significantly reduced hopelessness (*p* = 0.031) and improved self-concept (*p* < 0.001) over time, specifically among participants with fewer baseline assets (Kizito et al., 2025).

In South Africa, cash transfers combined with parenting support significantly improved mental health compared to cash transfers only (Cluver et al., 2019). Similarly, the ZAMFAM project in Zambia (Table 3), which provided integrated psychosocial, economic, and clinical support in Central Province, significantly reduced caregiver stigma (aPRR = 0.49, 95% CI: 0.28–0.88) compared to usual care, although there were no significant differences in depression levels (Rosen et al., 2021). A qualitative sub-study in Kenya, nested within the “Shamba Maisha” cluster RCT, involved agricultural training, provision of farming implements, and loans. Participants reported improved community empowerment, economic status, and food security, which contributed to better psychological well-being (Chi et al., 2025)

### 3.3. Pure Economic Interventions (Cash Transfers Only)

Interventions relying on conditional cash transfers showed inconsistent results. The impact of pure cash incentives conditional on clinic attendance, tested in RCTs in Tanzania, was mixed, showing a modest reduction in depression at month 12 in one trial (Chitle et al., 2024) but no significant effect on emotional distress in another at month six (Sheira et al., 2024). A non-randomized feasibility trial in Kenya combining cash transfers with lactation support found no significant between-group difference in depressive symptoms on a quantitative scale, despite qualitative reports of reduced financial stress and improved well-being (Tuthill et al., 2023). These results indicate that cash transfers alone may be insufficient for sustained mental health improvement among PLHIV in LMICs.

### 3.4. Mechanisms of Action (Subgroup Effects)

Qualitative and quantitative data point to reduced financial stress and increased hope/self-concept as key pathways through which economic empowerment interventions improve mental health. Qualitative studies revealed mechanisms: reduced food insecurity and economic self-sufficiency enhanced hope and psychological well-being (Chi et al., 2025). Subgroup effects favored women, with greater economic empowerment participation associated with reduced stigma, increased hope, and better well-being (Kellett & Gnauck, 2016). Full-time economic empowerment participants showed greatest benefits, suggesting dose–response effects.

A non-randomized feasibility trial in Kenya provided 10 monthly cash transfers and personalized lactation support compared to standard prevention of mother-to-child transmission care. In this study, the analysis found no significant between-group difference in depressive symptoms, though scores decreased in both groups. However, qualitative findings strongly suggested the intervention reduced financial stress and improved mental well-being, highlighting a discrepancy between the quantitative scale results and the lived experiences of the participants (Tuthill et al., 2023).

## 4. Discussion

This review suggests that economic empowerment interventions can be effective in improving mental health among vulnerable populations. We discuss our results based on the objectives, intervention design complexities, target population, and process model theory (Moore et al., 2015). The process design theory posits that the intervention creates a pathway that produces change. Only one included study evaluated mediation pathways using statistical mediation analysis. Through this theory’s pathways we identified improvements in family assets and employment as significant mediators of intervention effects. Evidence from the remaining studies was primarily qualitative or inferential, with participants reporting reduced financial stress, increased hope, and improved psychosocial well-being. Therefore, the proposed process pathway should be interpreted as a theoretically supported model rather than a consistently validated causal mechanism across the current evidence base.

Thus, our review refines the theory by highlighting that the pathway may be effectively activated by integrated interventions that combine economic resource provision with mechanisms for psychological resource development, such as peer support, mentorship, and goal-setting inherent in training, rather than by economic inputs alone.

Multi-component interventions that combined economic interventions and psychosocial or structural support programs were associated with mental health benefits compared to standalone interventions. These results add to the existing literature that consistently documents the correlation between economic status, food insecurity and mental health conditions among people living with HIV in LMICs (Ayano et al., 2020; Tsai et al., 2012; Watson et al., 2019).

In Cambodia, a KHANA livelihood program, which involved village savings/loan associations, skills training, and small cash grants, reduced the odds of depressive symptoms by 32% (Shimizu et al., 2016). Chi et al. (2025) suggested food security, not just income, may drive psychological benefits. The “Mlambe” intervention in Malawi concluded that economic empowerment and relationship skills, and incentivized joint savings accounts, showed significant reductions in depression and hopelessness compared to enhanced usual care (Van Doren et al., 2026). A cohort study in South Africa showed that cash transfers integrated with parenting skills support significantly improved mental health, whereas cash alone did not yield any significant improvement in mental health outcomes (Table 4 and Appendix A Table A3) (Cluver et al., 2019).

In contrast, the effect of pure cash incentives conditional on clinic attendance, tested in Tanzania, was mixed, showing a modest reduction in depression in one study but no significant effect on emotional distress in another (Chitle et al., 2024). This suggests that conditional short-term incentive-based cash transfer may not effectively address the chronic psychological challenges facing PLHIV. Improvements may partly reflect engagement in care rather than economic components alone (Chitle et al., 2024). Cash transfer alone may provide a temporary economic buffer but not a sustainable psychological resource needed to address structural vulnerabilities or maintain better mental health. A non-randomized feasibility trial in Kenya with cash transfers and personalized lactation support showed non-significant differences in mental health outcomes compared to standard prevention of mother-to-child transmission care (Tuthill et al., 2023).

All positive multi-component trials lasted for more than 12 months, while shorter cash-only trials showed null effects.

This suggests that economic empowerment intervention integrated with need-based psychological needs may be necessary for direct mental health outcomes for PLHIV, as suggested by different scholars (Nwogwugwu et al., 2025; Sheira et al., 2024; Wollburg et al., 2023).

The pathway through which these interventions improve mental health appears to operate via two interconnected mechanisms. We hypothesize that economic empowerment intervention (e.g., reducing financial stress by building assets) is the mediating pathway that affects positive mental health (e.g., increasing hope/self-concept) improvement among people living with HIV. The Suubi + Adherence RCT study in Uganda demonstrated that mental health improvement was mediated by economic stability (Cavazos-Rehg et al., 2021).

In Uganda, women who had combined access to ART, peer support, and economic empowerment intervention were qualitatively associated with greater hope, reduced HIV-related stigma, better mental health outcomes, and general well-being (Kellett & Gnauck, 2016). Participants in the Shamba Maisha agricultural program reported improved economic status and food security, contributing to enhanced psychological well-being (Chi et al., 2025). A non-randomized feasibility trial in Kenya found that despite no quantitative between-group difference in depressive symptoms, qualitative findings strongly suggested the intervention reduced financial stress and improved mental well-being (Tuthill et al., 2023).

All positive multi-component trials lasted more than 12 months, while shorter cash-only trials showed null effects. Thus, our review refines the process design theory: the pathway may be effectively activated by integrated interventions that combine economic resource provision with psychological resource development mechanisms such as peer support, mentorship, and goal-setting inherent in training rather than by economic inputs alone.

The benefits of these interventions were not uniform across populations. Our review points out that vulnerability-based targeting warrants further implementation research. Evidence indicates that participants with the lowest baseline assets, women, and adolescents receiving parenting support yielded higher scores of better mental health outcomes.

Our review found interventions more effective among lower-income and vulnerable groups. For example, Suubi + Adherence RCT in Uganda showed significant mental health improvement at 36 months (B = −0.45, *p* < 0.05), with family assets mediating 42–72% of effect (Table 2 and Table A2). Under the same RCT study that tested the effect of economic empowerment on viral suppression and mental health (depressive symptoms, hopelessness, and self-concept), no effect was shown on the full sample for any mental health outcomes (Table 2 and Appendix A Table A2). A secondary, pre-specified analysis of the same Ugandan RCT revealed important subgroup effects. While the intervention showed no significant effect on mental health outcomes in the full sample, effects amplified among adolescents with fewer baseline assets, reducing hopelessness (*p* = 0.031) and improving self-concept (*p* < 0.001) reiterating a greater effect on the more vulnerable adolescents (Table 2 and Appendix A Table A2).

Vulnerability-based targeting interventions revealed that parenting support and agricultural programs improved adolescent and community well-being in Kenya and South Africa (Table 2 and Table 3 and Appendix A Table A2) (Chi et al., 2025; Cluver et al., 2019).

Effects on women were also notable. For example, greater economic empowerment participation in women improved hope, reduced stigma, and contributed to well-being (Kellett & Gnauck, 2016). Additionally, there were subgroup benefits for low-asset participants, of which many were female-headed (Kizito et al., 2025), and 32% lower odds of depressive symptoms in the majority female sample that participated in savings/loan association, skills training, and small cash grants interventions in Cambodia (Shimizu et al., 2016).

### Limitations

Heterogeneity of the included studies stemming from different interventions (e.g., agricultural interventions, cash transfers, microfinance interventions), focus on different mental health conditions (e.g., depression, anxiety, hopelessness), different assessment and data collection tools, and different target populations hindered the possibility of conducting a meta-analysis for a precise overall effect estimates.

Due to heterogeneity, the reliance on narrative synthesis may be less objective compared to quantitative analysis.

The inclusion of non-randomized studies may introduce the risk of confounding and other biases. The observed associations cannot establish robustly tested causality because other necessary potential confounders were not controlled at the intervention or outcomes measurements.

Language and publication bias form part of this study’s limitations. This study included only peer-reviewed studies that were published in English. This may have excluded studies published in other languages.

Included studies had varying quality levels as seen in Table 1. The risk of bias in some studies may affect the strength and reliability of the findings.

We did not conduct a formal GRADE or GRADE-CERQual certainty assessment. This decision was made due to the heterogeneity of included study designs, interventions, and outcome measures, which precluded meta-analysis. While we acknowledge this as a limitation, we have provided transparent study-level quality appraisal using validated tools appropriate to each design (RoB2, ROBINS-I, CASP).

## 5. Conclusions and Policy Recommendations

Evidence from our study underscores that economic empowerment interventions may contribute to the improvement of the mental health of PLHIV in LMICs. However, the effectiveness of the intervention depends not only on simple cash transfer but a theoretically sound integrated approach. In our study, the process theory forms a base upon which the reduction in finance-based stress and the development of psychological resources not only improve mental health but also contribute to viral load suppression and retention among PLHIV in LMICs. It is upon these findings that we provide the following recommendations:

Design and fund multi-component economic (e.g., matched savings, small and medium enterprise support) and psychosocial (e.g., cognitive behavioral therapy, peer group support) interventions to efficiently and effectively improve the mental health of people living with HIV in low- and middle-income countries. Due to the chronic nature of HIV, the effects will be even better if the proposed interventions are long-term.

Integrate mental health into HIV services. Our study not only provides evidence of possible mental health improvement due to economic and psychosocial support, but also highlights these outcomes, including better viral load suppression and high retention rates.

Since economic hardship is a strong predictor of poor mental health outcomes, economic empowerment interventions to improve the mental health of people living with HIV need to be equitable by using means testing as elaborated by Kizito et al. (2025) or poverty-targeting to direct resources to those who are in the lowest poverty quintile.

Cash transfer as a strategy of improving the mental health of people living with HIV is showing mixed outcomes. We therefore recommend that program implementers and policy makers to design economic empowerment intervention that targets asset building, skills training, micro-enterprise support, matched savings, and co-financing of livelihood projects, among others.

We recommend that the involved parties invest in implementation science before scaling up economic and evidence-based interventions that target health improvements of PLHIV. Using a structured process model ensures the inclusion of crucial steps that may contribute to better health economic outcomes at both the individual and societal levels.

### Future Directions

Develop and evaluate multi-component, theory-driven interventions. Future research and intervention should be grounded on sound theoretical framework that combine economic empowerment models (e.g., asset building, skills training, microfinance lending), ecological support (e.g., social support, safety nets, enhanced health care access), and psychosocial support frameworks (e.g., cognitive behavioral therapy, peer support) that leads to mental health improvement among people living with HIV in low- and middle-income countries.

Implement long-term (preferably more than 12 months), longitudinal interventions, which are essential in determining the causal effect of different theories of sound economic and psychosocial support on the health of PLHIV. This can be mediated by better economic outcomes, ART adherence, viral load suppression, retention, and better mental health outcomes.

Our finding points out that vulnerability-based targeting warrants further implementation research. Evidence indicates that participants with the lowest baseline assets, women and adolescents receiving parenting yielded higher scores of better mental health outcomes.

Promote standardization and methodological rigor in the implementation of economic empowerment interventions that aim at improving the mental health of PLHIV in LMICs. This may involve standardization of the economic empowerment intervention packages, a minimum implementation period, standardized evaluation tools, and age- and gender-sensitive designs among others. This will necessitate future meta-analysis that will generate evidence for cost-effective interventions worth scaling up.

## Figures and Tables

**Table 1:** Results of quality appraisal and risk of bias of the included studies.

Study Authors (Year)	Study Design	Risk of Bias/Quality Assessment Tool	Quality/Risk Rating
Tuthill et al. (2023)	Intervention (Non-Randomized)	ROBINS-I (for non-randomized studies)	Low Risk
Cluver et al. (2019)	Cohort	ROBINS-I (for non-randomized studies)	Moderate Risk
Rosen et al. (2021)	Cohort	ROBINS-I (for non-randomized studies)	Moderate Risk
Chi et al. (2025)	Qualitative	CASP (Qualitative Checklist)	High Quality
Kellett and Gnauck (2016)	Qualitative	CASP (Qualitative Checklist)	High Quality
Cavazos-Rehg et al. (2021)	RCT (Individual)	RoB 2 (for randomized trials)	Some concerns
Shimizu et al. (2016)	Quantitative (Program Evaluation)	RoB 2 (for randomized trials)	Some Concerns
Kizito et al. (2025)	RCT (Individual)	RoB 2 (for randomized trials)	High quality
Sheira et al. (2024)	RCT (Individual)	RoB 2 (for randomized trials)	Some Concerns
Van Doren et al. (2026)	RCT (Individual)	RoB 2 (for randomized trials)	Some Concerns
Chitle et al. (2024)	RCT (Individual)	RoB 2 (for randomized trials)	Some Concerns

**Table 2:** Summary of included experimental and quasi-experimental studies.

Authors and Study Location	Study Objectives	Study Design: Intervention and Control	Outcome Effect Size	Summary of Outcomes	Other Outcomes
Kizito et al. (2025) Southern Uganda	Examine the impact of family-based economic empowerment on viral suppression (primary) and mental health (secondary).	Intervention (Suubi + Adherence): 24-month cluster RCT. Matched youth savings account, financial literacy training, microenterprise workshops. **Control:** Bolstered standard of care (adherence sessions, cartoon literature).	**Full Sample:** No significant main or interaction effects on mental health **Low-Asset Subgroup:** Significant improvement in hopelessness at Y3 MD = −1.19 ((95% CI: −2.00, −0.004), *p* = 0.004 and self-concept at Y3 MD = 2.50 ((95% CI: 0.48, 4.53), *p* = 0.016; and Y7 MD = 3.81 (95% CI: 0.76, 6.86), *p* = 0.014).	No mental health effect for the full sample. Significant improvements in hopelessness and self-concept for the most economically vulnerable adolescents.	Primary outcome (viral suppression) significantly improved at years 2, 3, and 4.
Shimizu et al. (2016) Cambodia	Examine impact of a livelihood program on depressive symptoms among people living with HIV (PLHIV).	**Intervention:** Quasi-experimental. KHANA livelihood program: Village savings/loan associations, skills training, small cash grants. **Control:** Non-participants from same health centers.	AOR: 0.68 (95% CI: 0.52–0.88); Propensity Score Matching ATT: T = −1.99.	Participation in the livelihood program was associated with 32% lower odds of depressive symptoms.	High baseline prevalence of depressive symptoms (59.2% total).
Cavazos-Rehg et al. (2021) Uganda	Examine mediating mechanisms between a family economic intervention and mental health of ALHIV.	**Intervention:** (Suubi + Adherence): Longitudinal cluster RCT. Child Development Accounts, workshops, peer mentorship. **Control:** Bolstered standard of care.	**Mental Health at 36 mo**: B = −0.45 (latent score combining depression, hopelessness and self-concept), β = −0.10 (Significant). **Mediation:** Family assets/employment was the only significant mediator (explained 42–72% of effect).	The intervention improved mental health, particularly at 36 months. The effect was primarily driven by improvements in family economic stability.	Other mediators (viral suppression, stigma, food security) were not significant.
Chitle et al. (2024) Tanzania	Assess if financial incentives for clinic attendance improve mental health among adult ART initiates.	**Intervention:** Two-arm cluster-RCT. Opportunity to earn 6 monthly cash incentives, conditional on clinic attendance. **Control:** Standard-of-care HIV primary care.	Depression at 12 mo (IPCW DiD): −5.5, pp (95% CI: −0.20, −10.8; *p* = 0.04).	A modest significant reduction in depression symptoms was found in the incentive arm at 12 months, but not at 6 months.	Both arms showed large improvements in mental health over time, attributed to ART initiation and care engagement.
Sheira et al. (2024) Tanzania	Evaluate impact of financial incentives on mental health of adults initiating ART.	**Intervention:** Three-arm RCT secondary analysis. Monthly conditional cash incentives (~$4.50 or $10) for clinic attendance. **Control:** Standard of care.	Diff-in-Diff (Emotional Distress): −2.7 pp (95% CI: −13.2, 7.7); not significant.	Cash incentives did not provide a statistically significant improvement in emotional distress over standard of care alone.	High baseline prevalence of mental health symptoms improved in both groups over time.
Tuthill et al. (2023) Kenya	Assess feasibility/acceptability of unconditional cash transfers + infant feeding support for WLHIV.	**Intervention:** Non-randomized feasibility trial. 10 monthly cash transfers + personalized lactation support. **Control:** Standard PMTCT care.	PHQ-9 at 6 mo PP: Coeff = −1.40 (95% CI: −4.51, 1.84), *p* = 0.38 (Not significant).	Quantitative analysis found no significant between-group difference in depressive symptoms, though scores decreased in both groups.	Qualitative findings strongly suggested the intervention reduced financial stress and improved mental wellbeing. Significant reduction in food insecurity.
Van Doren et al. (2026) Malawi	To evaluate the effects of an economic and relationship-strengthening intervention (Mlambe) on mental health outcomes (depression, stress, hopelessness) among couples with HIV and unhealthy alcohol use.	**Design:** Pilot randomized controlled trial (RCT) **Intervention:** Mlambe program (10 monthly sessions on economic empowerment and relationship skills, plus incentivized joint savings accounts). **Control:** Enhanced usual care (EUC), including brief alcohol counseling and standard HIV care.	**Depression:** Cohen’s d = 0.41–0.46; (*p* < 0.05). **Stress:** Cohen’s d = 0.52–0.54; (*p* < 0.05). **Hopelessness:** Cohen’s d = 0.26–0.29; (*p* < 0.05).	The Mlambe intervention resulted in statistically significant reductions in depression, stress, and hopelessness compared to the control group at both 10- and 15-month follow-ups. Women experienced significantly greater reductions in stress and hopelessness than men.	- High feasibility and acceptability of the intervention. - Reductions in alcohol use and improvements in ART adherence (primary outcomes reported elsewhere). - Qualitative data indicated enhanced hope and reduced interpersonal stress.

Note: All abbreviations in the table are described under the list of abbreviations section.

**Table 3:** Summary of included qualitative studies.

Authors and Study Location	Study Objectives	Study Design: Intervention and Control	Outcome Effect Size	Summary of Outcomes	Other Outcomes
Chi et al. (2025) Kenya	Examine participant perspectives on community-level effects (spillover) of an agricultural livelihood intervention.	**Intervention:** (“Shamba Maisha”): Qualitative sub-study nested in cluster RCT. Agricultural training, farming implements, and loan. **Control:** Standard care.	(Qualitative Study)	Participants reported improved community empowerment, economic status, and food security, which contributed to improved psychological well-being.	Themes included new leadership roles, women’s empowerment, and supporting vulnerable community members.
Kellett and Gnauck (2016) Uganda	To explore perceptions of HIV stigma among HIV-positive women with similar access to ART and peer support but varying levels of participation in economic empowerment programs.	**Design**: Qualitative focus group study. **Intervention/Exposure:** ART + HIV peer support groups + varying participation in economic empowerment programs (full-time, intermittent, none). **Control/Comparison:** Comparisons based on level of participation in economic empowerment programs. **Activities.** **LifeStitches:** Full-time sewing workshop providing income-generating skills, equipment, and market access (highest EE intensity). **NACWOLA:** National Community of Women Living with HIV/AIDS in Uganda; part-time crafts workshop providing counselling and peer support (medium EE intensity). **PMTCT:** Prevention of Mother-to-Child Transmission clinic peer support group; ART + peer support only, no EE program (comparison group). **EE:** Economic empowerment interventions.	(Qualitative Study)	Stigma persists (blame, uselessness, disclosure avoidance). ART improved health status, changing perceptions from “useless” to “useful.” Peer support provided psychological well-being and reduced fear. Economic empowerment enhanced self-sufficiency, social status, hope, and positive thinking. Greatest benefits observed among full-time workshop participants (LifeStitches).	HIV-negative infants through PMTCT enhanced self-confidence and family status. Full-time economic empowerment (LifeStitches) appeared to have synergistic effects with peer support in overcoming stigma.

Note: All abbreviations in the table are described under the list of abbreviations section.

**Table 4:** Summary of included cohort Studies.

Authors & Study Location	Study Objectives	Study Design: Intervention and Control	Outcome Effect Size	Summary of Outcomes	Other Outcomes
Cluver et al. (2019) South Africa	To test the UN Development Programme’s approach of “development accelerators” on achieving SDG-aligned targets among adolescents living with HIV.	**Design:** Prospective cohort study. **Intervention/Exposure:** Access to real-world provisions (e.g., parenting support, cash transfers, safe schools). **Control/Comparison:** No formal control group; comparisons based on level of access.	**Cash Transfer and Mental Health:** aOR = 1.10 (95% CI: 0.69–1.76; *p* = 0.70). **Parenting Support and Mental Health:** aOR = 2.13 (95% CI: 1.43–3.15; *p* < 0.0001). **Safe Schools and Mental Health:** aOR = 1.74 (95% CI: 1.30–2.34; *p* < 0.0001).	Cash transfers were not associated with improved mental health. Parenting support and safe schools were significant accelerators for good mental health and other SDG targets.	Cash transfers were associated with HIV care retention, school progression, and no emotional/physical abuse. Combinations of accelerators showed synergistic effects.
Rosen et al. (2021) Zambia	To evaluate the impact of the multi-component ZAMFAM Project on ALHIV and their caregivers.	**Design:** Prospective cohort study (non-randomized). **Intervention:** ZAMFAM Project (integrated psychosocial, economic, clinical support) in Central Province. **Control:** Usual care in Eastern Province. **Intervention:** Participation in monthly, hospital-linked “Kids Clubs” support groups. **Control:** No control group.	**Caregiver Stigma:** aPRR = 0.49 (95% CI: 0.28–0.88). **ALHIV Depression:** Not significant in adjusted analysis.	Integrated interventions improved economic and psychosocial outcomes for caregivers. Few significant direct health changes for adolescents.	For ALHIV: improvements in ART use, reduction in labor. For caregivers: reduced negative community attitudes, improved self-reported health.

Note: All abbreviations in the table are described under the list of abbreviations section.

## Data Availability

The original contributions presented in this study are included in the article. Further inquiries can be directed to the corresponding author.

## Abbreviations

AIDS	Acquired Immunodeficiency Syndrome
ALHIV	Adolescents Living with HIV
ART	Antiretroviral Therapy
aOR	Adjusted Odds Ratio
aPR	Adjusted Prevalence Ratio
aPRR	Adjusted Prevalence Rate Ratio
ATT	Average Treatment Effect on the Treated
BHS	Beck Hopelessness Scale
CASP	Critical Appraisal Skills Programme
CBT	Cognitive Behavioral Therapy
CDI	Child Depression Inventory
CES-D	Center for Epidemiologic Studies Depression Scale
CI	Confidence Interval
DiD	Difference-in-Difference
EE	Economic empowerment interventions.
GAD-2	Generalized Anxiety Disorder-2
HIV	Human Immunodeficiency Virus
HSCL-25	Hopkins Symptom Checklist-25
HSCL-D	Hopkins Symptom Checklist for Depression
IPCW	Inverse Probability of Censoring Weighting
IQR	Interquartile Range
LMICs	Low- and Middle-Income Countries
MD	Mean Difference
Mo	Month
N	Sample Size
NS	Not Significant
OVC	Orphans and Vulnerable Children
PHQ	Patient Health Questionnaire
PLHIV	People Living with HIV
pp	Percentage Points
PRISMA	Preferred Reporting Items for Systematic Reviews and Meta-Analyses
PROSPERO	International Prospective Register of Systematic Reviews
PSS	Perceived Stress Scale
PTSD	Post-Traumatic Stress Disorder
RCT	Randomized Controlled Trial
RoB 2	Risk of Bias 2 (Tool)
ROBINS-I	Risk Of Bias In Non-randomized Studies-of Interventions
SD	Standard Deviation
SDG	Sustainable Development Goals
SHG	Self-Help Group
TSCS	Tennessee Self-Concept Scale
UN	United Nations
VL	Viral Load
VSLA	Village Savings and Loan Association
WLHIV	Women Living with HIV
Y3	Year 3
NACWOLA	National Community of Women Living with HIV/AIDS
PMTCT	Prevention of Mother-to-Child Transmission.

## Appendix A

**Table A1:** Systematic characterization of intervention characteristics and outcomes.

Study	Intervention Category	Economic Components	Psychosocial Components	Mental Health Outcome(s)	Effect Direction	Population	Key Moderators Identified
Kizito et al. (2025)	Multi-component	Matched savings, financial literacy	Peer mentorship	Depression, hopelessness, self-concept	+ (subgroup only)	ALHIV	Baseline assets
Shimizu et al. (2016)	Multi-component	VSLA, cash grants, skills training	Not specified	Depression	+	PLHIV	None reported
Cavazos-Rehg et al. (2021)	Multi-component	Child development accounts	Peer mentorship	Latent MH construct	+	ALHIV	Family assets (mediator)
Van Doren et al. (2026)	Multi-component	Joint savings, incentivized accounts	Relationship skills	Depression, stress, hopelessness	+	HIV+ couples	Gender
Cluver et al. (2019)	Multi-component	Cash transfer	Parenting support	Composite MH	+ (combined only)	ALHIV	Parenting support
Chi et al. (2025)	Livelihood/Ag	Agricultural training, loans	None specified	Well-being (qual)	+ (qual only)	PLHIV	Food security
Tuthill et al. (2023)	Multi-component	Cash transfers	Lactation support	Depression	0 (quant), + (qual)	WLHIV	Financial stress
Chitle et al. (2024)	Cash only	Conditional cash incentives	None	Depression, anxiety	+/−	PLHIV	None
Sheira et al. (2024)	Cash only	Conditional cash incentives	None	Emotional distress	0	PLHIV	None
Rosen et al. (2021)	Multi-component	Economic strengthening (unspecified)	Kids Clubs, psychosocial	Depression	0	ALHIV + caregivers	Stigma (reduced)

Notes: + = positive/ significant; 0 = null; +/− = mixed; qual = qualitative; quant = quantitative. For qualitative studies, “+” indicates that participants consistently reported positive perceived effects rather than statistically significant quantitative effects.

**Table A2:** Detailed summary of included experimental and quasi-experimental studies.

Authors and Study Location	Study Objectives	Population at Baseline (N, % Female, Age)	Population at Endline (N, % Female, Age)	Tools for Assessment	Summary of Outcomes
Kizito et al. (2025) Southern Uganda	Examine the impact of family-based economic empowerment on viral suppression (primary) and mental health (secondary).	N = 702; 56.4% Female; Mean Age: 12.43 years (SD = 1.98, Range 10–16). All adolescents living with HIV (ALHIV) on ART.	7-year follow-up; Attrition: 18% over 7 years.	Depression: Child Depression Inventory (CDI), CES-D; Hopelessness: Beck’s Hopelessness Scale; Self-Concept: Tennessee Self-Concept Scale.	No mental health effect for the full sample. Significant improvements in hopelessness and self-concept for the most economically vulnerable adolescents.
Shimizu et al. (2016) Cambodia	Examine impact of a livelihood program on depressive symptoms among people living with HIV (PLHIV).	N = 685 (Intervention: 357, Control: 328); 68.3% Female; Mean Age: 44.0 years (SD = 8.3). All PLHIV (97.8% on ART).	Single post-intervention assessment; Minimal attrition (1 excluded/group).	Depressive Symptoms: Hopkins Symptom Checklist-25 (HSCL-25).	Participation in the livelihood program was associated with 32% lower odds of depressive symptoms.
Cavazos-Rehg et al. (2021) Uganda	Examine mediating mechanisms between a family economic intervention and mental health of ALHIV.	N = 702; 56% Female; Age: 10–16 years. All ALHIV on ART.	24, 36, 48-month follow-ups; Low attrition.	Latent Mental Health Construct from Beck Hopelessness Scale (BHS), Children’s Depression Inventory (CDI), Tennessee Self-Concept Scale (TSCS).	The intervention improved mental health, particularly at 36 months. The effect was primarily driven by improvements in family economic stability.
Chitle et al. (2024) Tanzania	Assess if financial incentives for clinic attendance improve mental health among adult ART initiates.	N = 1990; 59.5% Female; Mean Age: 36.7 years (SD = 11.5). Adults newly initiating ART.	6 and 12-month follow-ups; Used IPCW for missing data.	Depression: PHQ-2; Anxiety: GAD-2.	A modest significant reduction in depression symptoms was found in the incentive arm at 12 months, but not at 6 months.
Chi et al. (2025) Kenya	Examine participant perspectives on community-level effects (spillover) of an agricultural livelihood intervention.	N = 70 (Int: 40, Con: 30); 60% Female; Mean Age: ~39.6 years. PLHIV with food insecurity/malnutrition.	End of intervention; Qualitative interviews.	(Qualitative Study)	Participants reported improved community empowerment, economic status, and food security, which contributed to improved psychological well-being.
(Sheira et al. (2024) Tanzania	Evaluate impact of financial incentives on mental health of adults initiating ART.	N = 530; 62.3% Female; Mean Age: 36.1 years (SD = 10.2). Adults initiating ART.	6-month follow-up; Used multiple imputation.	Emotional Distress: Hopkins Symptom Checklist-25 (HSCL-25).	Cash incentives did not provide a statistically significant improvement in emotional distress over standard of care alone.
Tuthill et al. (2023) Kenya	Assess feasibility/acceptability of unconditional cash transfers + infant feeding support for WLHIV.	N = 40 (Int: 20, Con: 20); 100% Female; Mean Age: ~28.3 years. Pregnant/postpartum women living with HIV.	6 months postpartum; 100% retention.	Depression: PHQ-9; Stress: PSS-10.	Quantitative analysis found no significant between-group difference in depressive symptoms, though scores decreased in both groups.
Van Doren et al. (2026) Malawi	To evaluate the effects of an economic and relationship-strengthening intervention (Mlambe) on mental health outcomes (depression, stress, hopelessness) among couples with HIV and unhealthy alcohol use.	**N:** 156 individuals (78 couples) **Female:** ~50% (equal number of male and female partners) **Mean Age:** 43.4 years (SD = 10.2; range: 21–80)	**N:** >96% retention, so approximately 150 individuals retained at both 10- and 15-month follow-ups. **Female:** ~50% (retention was high and equal by arm) **Age:** Not separately reported at endline.	**Depression:** 10-item Center for Epidemiologic Studies Depression Scale (CES-D) **Stress:** 10-item Perceived Stress Scale (PSS) **Hopelessness:** 20-item Beck Hopelessness Scale (BHS)	The Mlambe intervention resulted in statistically significant reductions in depression, stress, and hopelessness compared to the control group at both 10- and 15-month follow-ups. Women experienced significantly greater reductions in stress and hopelessness than men.
Kellett and Gnauck (2016) Uganda	To explore perceptions of HIV stigma among HIV-positive women with similar access to ART and peer support but varying levels of participation in economic empowerment programs.	**N = 54** (LifeStitches full-time: n = 18; NACWOLA part-time: n = 18; PMTCT no EE: n = 18). 100% Female. **Mean Age:** LifeStitches 38 yrs; NACWOLA 43 yrs; PMTCT 26 yrs. **Mean Education**: LifeStitches Grade 6; NACWOLA Grade 3; PMTCT Grade 5. All HIV-positive women on ART.	**Single assessment; qualitative focus groups.**	Semi-structured focus group guide (30 stem questions + probes) covering: HIV/AIDS knowledge, transmission, testing/treatment, discrimination. Analysis: Thematic analysis with focused coding.	Stigma persists (blame, uselessness, disclosure avoidance). ART improved health status, changing perceptions from “useless” to “useful.” Peer support provided psychological well-being and reduced fear. Economic empowerment enhanced self-sufficiency, social status, hope, and positive thinking. Greatest benefits observed among full-time workshop participants (LifeStitches)

Note: All abbreviations in the table are described under the list of abbreviations section.

**Table A3:** Detailed summary of included cohort studies.

Authors & Study Location	Study Objectives	Population at Baseline (N, % Female, Age)	Population at Endline (N, % Female, Age)	Tools for Assessment	Summary of Outcomes
Cluver et al. (2019) South Africa	To test the UN Development Programme’s approach of “development accelerators” on achieving SDG-aligned targets among adolescents living with HIV.	**N:** 1063 **% Female:** 55% **Age:** Mean 13.8 years (Range: 10–19). **Other:** All on ART; 15% had good mental health at baseline.	**N:** 994 (94% retention) **% Female and Age:** Not specified, assumed similar.	**Mental Health:** Composite of no depression (Child Depression Inventory), anxiety (Revised Children’s Manifest Anxiety Scale), or PTSD (Child PTSD Checklist).	Cash transfers were not associated with improved mental health. Parenting support and safe schools were significant accelerators for good mental health and other SDG targets.
Rosen et al. (2021) Zambia	To evaluate the impact of the multi-component ZAMFAM Project on ALHIV and their caregivers.	**N:** 544 dyads. **% Female (ALHIV):** 58.5%; (Caregivers): 87.9% **Age (ALHIV):** Median 11 yrs; (Caregivers): Median 43 yrs. **% Female:** 52.9% **Age:** Range 8–29 years.	**N:** 494 dyads (91% retention). **% Female and Age:** Not specified.	**Caregiver Stigma:** Items from MEASURE Evaluation’s OVC Survey Toolkit.	Integrated interventions improved economic and psychosocial outcomes for caregivers. Few significant direct health changes for adolescents.

Note: All abbreviations in the table are described under the list of abbreviations section.
